# Anxiety and Depressive Traits in the Healthy Population Does Not Affect Spatial Orientation and Navigation

**DOI:** 10.3390/brainsci13121638

**Published:** 2023-11-26

**Authors:** Isma Zafar, Ford Burles, Lila Berger, Michael McLaren-Gradinaru, Adam Leonidas David, Inderpreet Dhillon, Giuseppe Iaria

**Affiliations:** Canadian Space Health Research Network, NeuroLab, Department of Psychology, Hotchkiss Brain Institute, Alberta Children’s Hospital Research Institute, University of Calgary, Calgary, AB T2N 1N4, Canada

**Keywords:** cognition, cognitive map, getting lost, orientation, path integration

## Abstract

The ability to navigate and orient in spatial surroundings is critical for effective daily functioning. Such ability is perturbed in clinically diagnosed mood and anxiety disorders, with patients exhibiting poor navigational skills. Here, we investigated the effects of depression and anxiety traits (not the clinical manifestation of the disorders) on the healthy population and hypothesized that greater levels of depression and anxiety traits would manifest in poorer spatial orientation skills and, in particular, with a poor ability to form mental representations of the environment, i.e., cognitive maps. We asked 1237 participants to perform a battery of spatial orientation tasks and complete two questionnaires assessing their anxiety and depression traits. Contrary to our hypothesis, we did not find any correlation between participants’ anxiety and depression traits and their ability to form cognitive maps. These findings may imply a significant difference between the clinical and non-clinical manifestations of anxiety and depression as affecting spatial orientation and navigational abilities.

## 1. Introduction

Effective spatial navigation is essential for everyday functioning, allowing a person to maintain a sense of direction and location while exploring their spatial surroundings [1], heading towards a favorite location, or simply walking around the neighborhood. This complex behavior mostly relies on the spatial knowledge and perceptual cues (i.e., landmarks) available in the environment that allow individuals to plan and execute a path to a target location while mentally updating their position and orientation in their minds [1]. One can appreciate the importance of this cognitive function, which is critical for socially interacting with the outside world, as well as for more basic needs, such as searching for food or avoiding danger [2]. Failing to make use of effective orientation and navigational skills could lead to upsetting consequences [1].

In order to navigate effectively within the environment, humans tend to form and make use of cognitive maps. Cognitive maps are mental representations of the environment critical for reaching any spatial location from somewhere else, even using routes not previously explored [3]. These (mental) maps allow individuals to store information about the location of environmental landmarks and, importantly, their relationship with one another, which allows them to get to any place from anywhere within the environment [3].

Early studies in rodents and more recent work in humans highlight the role of the hippocampus (i.e., a region in the medial temporal lobe) as being critical for the formation and use of cognitive maps. Lesion studies in rodents found that hippocampal damage results in the hindrance of spatial memory and navigational skills [4]. More specifically, hippocampal “place cells” are involved in the formation of cognitive maps [3] by firing at given spatial locations of the animal within the environment [5]. Place cells have also been documented in humans by recording neural activity in epileptic patients [6], which was possible through electrodes implanted in the patient’s brains to record epileptic seizures. As in rodents, also in this human study, place cells fired in response to specific regions of an environment and function to provide the foundation for an internal representation of the spatial layout, or a cognitive map [3,7]. In sum, evidence from both rodent and human studies supports the crucial role of the hippocampus (combined with inputs from the parahippocampal region) in forming cognitive maps essential for orientation and navigation [3,7].

Intriguingly, the role of the hippocampus is also interconnected with anxiety and depression disorders [8]. In fact, depression has been definitively related to structural abnormalities of the hippocampus, including a decreased hippocampal volume in patients suffering from depression [9]. Likewise, neuroimaging studies have also reported hippocampal abnormalities in various forms of anxiety [10], confirming the existence of a neurological overlap between the role of the hippocampus in spatial navigation and its affected function in mood disorders. A study by Mueller and colleagues [10] observed navigational deficits in children with anxiety disorder who made more heading direction errors and had worse accuracy in completing a spatial orientation task [10] as compared to a group of non-anxious children. These findings are consistent with early literature in rodents showing that the hippocampus is implicated in both anxiety-related behaviors as well as spatial-related behavior [11]. Moreover, a research study by Gould and colleagues [12] analyzed performance on a virtual spatial navigation task in depressed individuals to assess spatial memory and navigational skills; in this study, patients performed significantly worse than healthy participants as assessed by the number of locations found in a virtual town, a deficit in performance that the researcher attributed to the patients’ abnormal hippocampal functioning [12,13]. In summary, individuals diagnosed with depression displayed impaired navigational abilities, and children with anxiety also exhibited hindered spatial orientation, suggesting that the presence of clinically diagnosed anxiety and depression may be related to spatial navigation and orientation deficits.

The past literature portrayed the impact of anxiety and depression on navigational deficits as the same hippocampal structure is critically involved in both functions [10,12]. However, a prominent gap in the literature is that most, if not all, research has been conducted on patients with clinical manifestations of mood disorders (particularly anxiety and depression). This focus on clinical cases does not account for the possibility that mood, as the most common and shared aspect of human existence [14], could also be related to the human ability to orient. Aside from the clinically diagnosed condition of depression and anxiety, mood is also a common and shared aspect of human existence [14], with, interestingly, individuals subconsciously selecting particular music to change or maintain their mood [15]. There is extensive evidence demonstrating positive affect (PA) and negative affect (NA) being the two dominant dimensions of self-reported mood [14]. NA is known to be a general factor of subjective distress and contains an array of negative mood states; this entails fear, anxiety, hostility, and mood states related to depression, such as sadness [14]. Conversely, PA reflects one’s level of pleasurable engagement with the environment. Studies conducted on healthy subjects showed that anxiety is essentially a state of high NA, but depression is a mixed state of high NA and low PA [16]. Given a significant neurological overlap between anxiety, depression, and spatial orientation, one may hypothesize that the two facets of anxiety and depression traits could be related to an individual’s ability to orient and navigate in the spatial surroundings.

To test this hypothesis, we asked participants to perform a series of spatial orientation tasks in virtual (video game-like) environments and complete the State-Trait Anxiety Inventory (STAI) and the Patient Health Questionnaire (PHQ-9) to assess anxiety and depression traits [17,18]. As predicted by previous evidence showing that individuals diagnosed with depression [12] and anxiety disorder [10] exhibit impaired spatial navigation, we hypothesized that a significant correlation exists between anxiety and depression traits and spatial orientation skills; specifically, we expected that higher levels of anxiety and depression traits would result in poorer performance on a spatial orientation task assessing the ability to form cognitive maps.

## 2. Materials and Methods

### 2.1. Participants

We recruited 1237 healthy participants (696 females and 541 males) from the University of Calgary’s Research Participation System (RPS) and the NeuroLab’s online testing platform (www.gettinglost.ca (accessed on 16 January 2023)). The sample included participants with an age range of 18 to 65 years and a mean age of 25 years. Participants reported no neurological conditions, brain injuries, or cognitive complaints. This study was approved by the research ethics board of the University of Calgary.

### 2.2. Procedure

After providing demographic information, participants were asked to perform a battery of spatial orientation tasks from the NeuroLab’s online testing platform (www.gettinglost.ca (accessed on 16 January 2023)). Then, participants were asked to complete two questionnaires assessing for anxiety and depression traits. Table 1 reports the number of participants completing each task and each questionnaire.

### 2.3. Materials/Tasks

#### 2.3.1. Spatial Orientation Tasks

**Figure 1:**
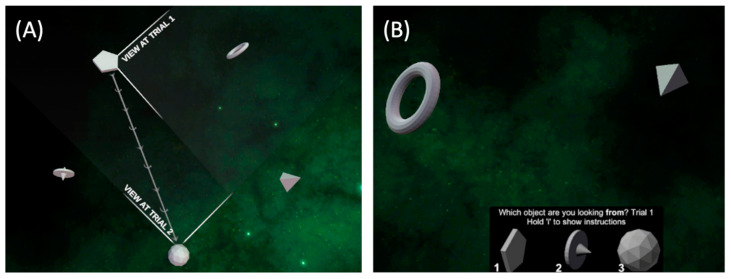
(**A**) The five objects in a pentagon shape in the Spatial Configuration Task from a bird’s eye view and the represented perspective at two subsequent trials. Participants never see the task from this perspective. (**B**) Participants were asked to determine which object shown at the bottom of the screen they thought they were looking from while viewing the two other objects in space.

**The Spatial Configuration Task** was used to measure the ability of participants to form a mental representation of the environment and the spatial relationships between the elements within it (i.e., cognitive maps) [19]. The task consisted of 60 trials performed in a space-like virtual environment in which five objects were randomly placed in a pentagon shape (see Figure 1A). In each trial, participants were shown a first-person viewpoint of the camera sitting on one of the objects while viewing two other objects; they were required to identify which object the camera was placed on by selecting one of the three objects displayed at the bottom of the screen (see Figure 1B). During every trial, the camera moved from one stationed object to a different one, allowing the participants to make (with time) a mental map of the location of all five objects in the environment. We measured the number of correct responses that participants provided throughout the entire task.

**Figure 2:**
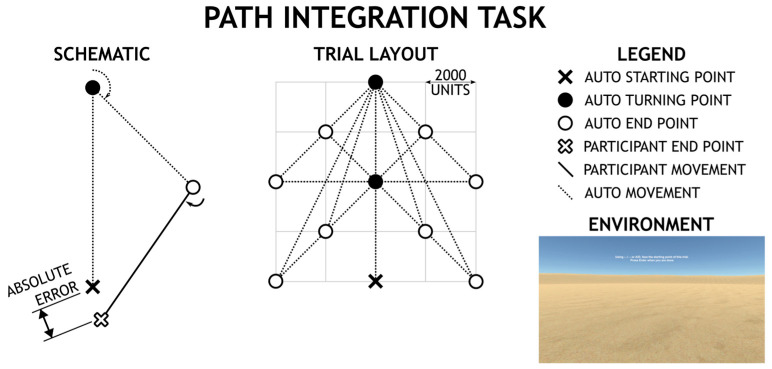
The figure shows a schematic drawing of the Path Integration Task in which participants use the information processed along the two segments of the triangular path (dotted lines) to judge the distance to be traveled along the third segment (dotted line). The figure also shows the participants’ view as it appears in the virtual environment in which no visual relevant information (e.g., landmarks) is available to solve the test. The textured floor creates the optic flow information that enables the processing of traveled distances and rotations.

**The Path Integration Task** measured the ability to navigate without any visual cues in a simple environment. This task requires participants to keep track of a person’s current position relative to a home location while locomoting through a series of two turns on a route designed on a triangle (non-visible) path [20] (see Figure 2). The task assesses the ability to integrate a spatial displacement using optic flow [20]; unlike real-world navigation where the vestibular system is used to regulate the integrative processing of optic flow, research has shown that path integration is possible through visual processing of optic flow alone through the virtual task [20]. The task consisted of 16 trials, and in each trial, participants had a first-person viewpoint of the camera moving forward, making a (left or right) turn, and then continuing to move forward, completing two sides of an imaginable triangle. At the end of these two movements, participants are asked to return directly to the starting point of the trial (closing the imaginable triangle). Participants first respond by rotating to face the starting point of the trial, then once they have locked in their heading, they are prompted to translate towards the starting point of the trial. Performance is recorded in the absolute Euclidean distance between the actual start position and the subject’s indicated position in virtual units (roughly equivalent to millimeters). A higher absolute error relates to worse performance. This task was based on the real-world task used by Wiener and colleagues [21].

**Figure 3:**
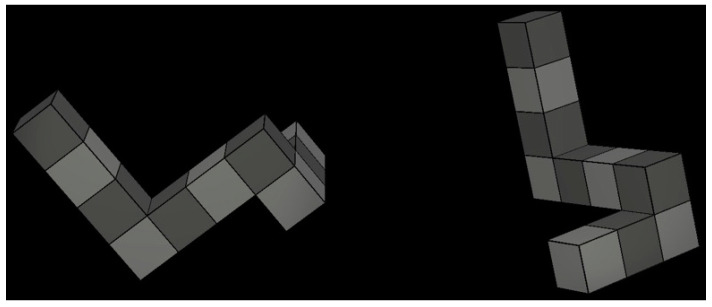
One sample trial of the Mental Rotation Task where participants are required to identify whether the two images side by side are the same or different from each other.

**The Mental Rotation Task** was used to measure the individual’s ability to perform mental manipulations of three-dimensional objects in space [22]. The task consisted of 80 trials. In each trial, participants were presented with two objects side by side and asked whether they were the same or differed from each other (see Figure 3). The objects were shown in varying spatial orientations. Participants were required to mentally rotate the objects to determine whether or not they were the same. We measured the number of correct responses that participants provided throughout the entire task.

**Figure 4:**
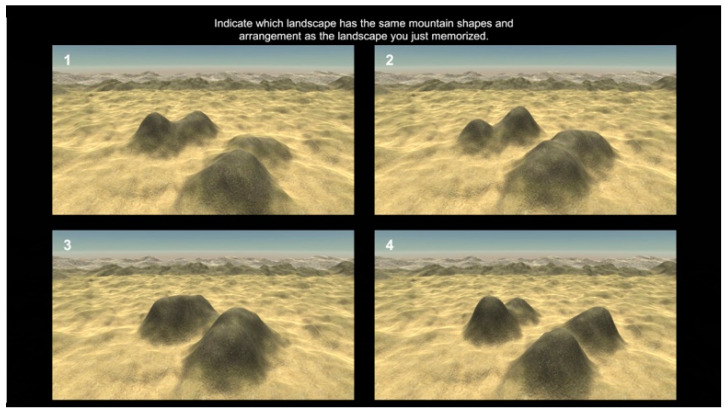
This displays the screen where participants are asked to recall the original picture and choose it from the four options displayed in the Four Mountains Task.

**The Four Mountains Task** measured the ability of the participants to recognize a scene from a different perspective (i.e., perspective-taking ability) [23]. The task consisted of 20 trials. In each trial, participants were presented with a simple scene of four mountains for eight seconds; afterward, they had to recall this scene and choose it from four options (see Figure 4), one of which depicted the original scene from a different perspective. We measured the number of correct responses that participants provided throughout the entire task.

#### 2.3.2. Mood Questionnaires

**The State-Trait Anxiety Inventory** (STAI-6) assessed participants’ current (state) and general (trait) anxiety symptoms [17]. The scale consisted of six items (e.g., Item one: “I feel calm”; Item two: “I am tense”), which were scored from 0 (not at all) to 3 (very much), providing a 0 to 18 total severity score. Higher scores implied an increased level of anxiety.

**The Patient Health Questionnaire** (PHQ-9) assessed an individual’s level of depression traits [18]. The scale consisted of nine items (e.g., Item one: “Little interest or pleasure in doing things”; Item two: “Feeling down, depressed, or hopeless”), which were scored from 0 (not at all) to 3 (nearly every day), providing a 0 to 27 total severity score. Higher scores implied an increased level of depressive symptoms.

### 2.4. Statistical Analysis

We used SPSS (IBM SPSS Statistics for Windows, Version 28.0. Armonk, NY: IBM Corp. Released 2021) to perform eight bivariate correlations. Bivariate correlations were used to determine if there was a statistically significant linear relationship between the variables and to determine the direction and the strength of the correlation. We correlated depression traits as assessed by the PHQ-9 questionnaire with each of the four spatial tasks (Spatial Configuration Task, Path Integration Task, Mental Rotation Task, and the Four Mountains Task). Similarly, we correlated anxiety traits as assessed by the STAI questionnaire with each of the four spatial tasks.

## 3. Results

We first looked at the correlation between the anxiety questionnaire and the tasks. The bivariate correlation revealed a non-significant correlation between the participants’ anxiety questionnaire score and their performance on the Four Mountains Task (*r*(166) = −0.04, *p* = 0.570), the Spatial Configuration Task (*r*(205) = −0.04, *p* = 0.549), the Mental Rotation Task (*r*(164) = −0.04, *p* = 0.653), and the Path Integration Task (*r*(159) = 0.01, *p* = 0.903) (Table 2). These results suggest that the anxiety traits of our healthy individuals have no direct relationship with a variety of skills important for spatial orientation and navigation, such as the ability to form cognitive maps, mentally rotate objects, track movement in space, and recognize places from a different perspective. These finds are not in line with what we have expected and hypothesized.

**Figure 5:**
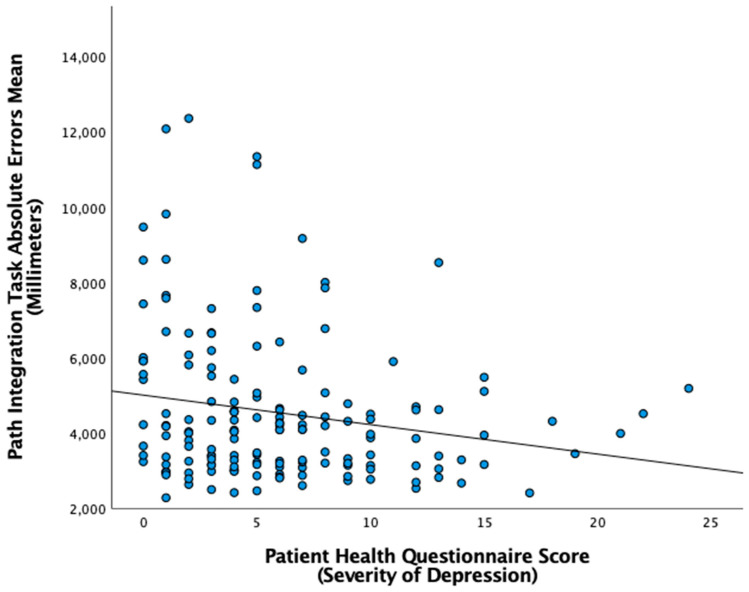
Path Integration Task Absolute Errors Mean by Patient Health Questionnaire Scores (PHQ-9). Path Integration Task recorded the error rate of the participant in reaching the initial (target) starting position; hence, higher absolute error (millimeters) relates to the worst performance on the task. PHQ-9 assessed the severity of depression with the following breakdown: minimal (0–4 score), mild (5–9 score), moderate (10–14 score), moderately severe (15–19 score), and severe (20–27 score).

Then, we looked at the relationship between participants’ depression traits and their performance at our tasks. The bivariate correlation revealed a non-significant correlation between the depression questionnaire score and the performance on the Four Mountains Task (*r*(167) = −0.07, *p* = 0.374), the Spatial Configuration Task (*r*(207) = −0.01, *p* = 0.857), and the Mental Rotation Task (*r*(166) = −0.03, *p* = 0.709) (Table 3). This suggests that participants’ depression traits are not related to their ability to form cognitive maps, mentally rotate objects, or recognize places from a different perspective. On the other hand, we found a significant correlation between participants’ depression questionnaire scores and their performance at the path integration task (*r*(160) = −0.19, *p* = 0.015); in this specific case, better performance correlated with higher (worse) depression trait (see Table 3 and Figure 5). This result is of particular interest since it suggests that high traits of depression may be related to a better performance in tracking one’s movement in order to locate a starting location.

## 4. Discussion

In this study, we investigated the relationship between mood and spatial navigation in healthy individuals. We utilized a battery of spatial tasks to assess participants’ navigational abilities. Specifically, we used the Spatial Configuration Task [19] to evaluate the ability of the individuals to form mental representations (cognitive maps) of the environment and examined how depression and anxiety traits would affect this important spatial ability. In addition, we investigated the relationship between depression and anxiety traits and spatial skills that are also important for spatial navigation: these tests included measures of the ability to orient without relying on visually relevant landmarks (i.e., the path integration task), the ability to perform simple mental rotations (i.e., the mental rotation task), and the ability to recognize a scene from a different perspective (i.e., four mountains task). Due to the documented evidence of impaired spatial navigational abilities in patients affected by major depressive and anxiety disorders [10,12], we hypothesized that healthy participants with high (but non-clinical) levels of anxiety or depression traits would have a poor ability to form cognitive maps. Contrary to our hypothesis, the results revealed no significant correlations between individuals’ depression and anxiety traits and the ability to form cognitive maps.

The lack of significant correlations between depression and anxiety traits and the ability to form cognitive maps may suggest a key distinction between the clinical versus the non-clinical (i.e., trait) manifestations of depression and anxiety. In fact, when a major depressive disorder is diagnosed, patients perform poorer than healthy individuals on a novel virtual reality measure of spatial memory [12]; this may not be the case when accounting for depression traits in healthy individuals, as we reported in our study. This difference in clinical and non-clinical traits in reference to spatial navigational skills could be explained by the neurological mechanism (specifically the hippocampal function) involved in both depression and spatial navigation. Depressed patients have exhibited abnormal hippocampal functioning inferred from structural abnormalities [9], causing dysfunction in the array of cognitive, affective, and behavioral abilities [13]. Although the hippocampus is an important structure responsible for spatial navigation [24], it is possible that a non-clinical (trait) manifestation of depression would not imply a significant alteration of its function, or alteration of other brain regions critically involved in spatial orientation.

Previous studies also reported patients with clinical anxiety disorder having overall impaired spatial performance. In fact, Mueller and colleagues [10] showed that anxious children made more heading directional errors and had more spatial orientation deficits than controls. Our findings reporting no significant correlation between anxiety traits and the ability to form cognitive maps may suggest that there may be a significant difference between the clinical and non-clinical manifestations of the anxiety disorder. The argument made above in reference to major depressive disorder, the inconsistency of spatial navigation impairments between clinical populations and healthy individuals with traits of a neurological condition could be due to the severity of the disorder in clinical conditions that affect the structure and functioning of selective neurological mechanisms involved in spatial navigation. Given that traits did not affect navigational abilities in our study, one could argue that this may be due to the less severe non-clinical symptoms as compared to the clinical ones [25]. In sum, clinical traits could imply changes in the neurological mechanisms that would eventually interfere with the proper functioning of brain regions involved in spatial orientation, whereas non-clinical traits may not.

The performance of the participants at the other spatial tasks replicated the null finding reported above: the ability to mentally rotate objects and recognize a place from a different perspective did not seem to be related to the anxiety and depression traits of the individuals. Surprisingly, however, individuals with higher depression traits performed better at the path integration task, which assessed the ability to integrate perceived self-motion over time using a triangle completion task [21]. A key distinction of the path integration task from the other spatial tasks utilized in our study is its usage of a simple environment; the task is indeed set up in an empty desert-like environment without any distinctive landmarks [26]; it is possible that since the environment was simplistic and less resembling to real-life, it involved neurological structures (and other cognitive functions) different from the higher-order ones involved in forming and using cognitive maps. A task such as the path integration task is indeed very different than the skills required while navigating in real surroundings, which are much more complex [27] and require various information processing systems, including the vestibular system utilized for motion detection [28] and update of body information by kinesthesis [29].

However, there are a handful of findings that suggest that in some domains, individuals with major depressive disorder (MDD) show alterations in a handful of different visual processing domains. For instance, researchers have reported decreased retinal and cortical sensitivity to visual contrast in individuals with MDD [30,31,32,33]. Of particular relevance to the path integration task is the results reported by Golomb and colleagues [34] in which they found that patients with MDD had enhanced visual motion perception, as explained by some dysfunction of cortical GABA in the MDD population. To elaborate, a benefit of reduced GABA in MDD individuals leads to enhanced processing of high-contrast large stimuli [34]. Rampacher and colleagues [35] also demonstrated that unlike other disorders (ex. obsessive–compulsive disorder), MDD patients with high depressive traits did not demonstrate any difficulties in their visual organization. However, many of these aforementioned studies use cycling gabor patch stimuli, which limits their interpretability in the context of the more complex visual flow field processing needed to accurately perform the path integration task. That being said, the brain regions known to be involved in processing egocentric-motion-compatible visual flow, such as the middle temporal visual area [36], have been shown to exhibit changed network functioning in MDD [37]. This cluster of findings leads us to speculate that depression-related modulations to visual and cortical motion processing are the mechanism responsible for the positive association between depressive symptoms and path integration accuracy. Future research could more clearly investigate the higher-order visual processing effects seen in depression to better understand if the low-level visual and cortical effects (like those identified by Golomb and colleagues [34]) are primarily responsible for the path integration processing differences we observed here.

There are a few limitations that should be noted in our study. First, we assessed the ability to navigate on a large scale by using virtual environments; while testing spatial skills in a virtual environment is a very convenient and efficient approach since it allows control of the information available in the environment, it does not account for the multiple factors present in real-life navigation. For example, it disregards the vestibular, kinesthetic, and proprioceptive sensory experiences that function simultaneously while navigating in real life. This is important as Hegarty and colleagues [29] indicated that the internal representation of an environment (i.e., a cognitive map) is encoded through various sensory inputs, including vision, vestibular cues, kinesthesis, and motor efference. Conducting our study in a virtual environment comes at the expense of losing other valuable information. Concerning our findings in particular, this limits the interpretability of these findings in the context of naturalistic navigation, as the performance differences we observed here may be mitigated or otherwise affected by multisensory integrative processes present in ecologically valid navigation and orientation. Due to these circumstances, future research should focus on testing spatial navigation and mood in an ecological environment or with research paradigms (such as electroencephalography) that are more able to separate effects at perceptual and cognitive levels. Another limitation of our study is the online nature of our testing, which did not allow us to monitor some participants while they were performing the tasks. This could be a problematic factor in our study, given that the participants’ level of focus can vary, and distractions can also hinder their performance. Prospective research can overcome this limitation by testing participants in a controlled laboratory setting. This will help reduce distractions and any confusion regarding task performance, allowing participants to perform at their best [38]. Lastly, some participants might have experience simulator sickness while performing the tasks in the virtual environment, which could have affected performance [39,40,41]; simulator sickness is quite similar to motion sickness, with the distinction that the participant receives optic flow but no body movement [39], and this could cause disturbances in the oculomotor system and perception [40]. Future research could evaluate and monitor participants’ simulator sickness to account for this variable in the data analyses.

## 5. Conclusions

In conclusion, our study provides evidence of no significant correlations between anxiety and depression traits and the ability to form cognitive maps in healthy individuals. This main finding is not consistent with the spatial navigation impairments reported in patients affected clinically by anxiety and depression. This seems to suggest that there may be a significant difference between the clinical versus the non-clinical manifestations of a disorder in the way it affects spatial orientation and navigation. Future studies could assess spatial orientation skills in ecological surroundings (addressing most of the limitations of our study) in both participants with clinical and non-clinical traits of depression and anxiety to confirm that spatial orientation skills are indeed affected only by the presence of clinically diagnosed traits. Further investigations could then combine behavioral findings with neuroimaging data to evaluate the functional and structural integrity of brain regions that are known to be critical for spatial orientation and navigation; this approach would provide the opportunity to test the hypothesis that affected spatial orientation skills in individuals with clinical traits of anxiety, and depression are caused by changes in neurological mechanisms rather than the presence of those traits in the healthy population.

## Figures and Tables

**Table 1:** Number of Participants Completing each Task (Four Mountains Task, Spatial Configuration Task, Mental Rotation Task, and Path Integration Task) and Questionnaire (State-Trait Anxiety Inventory and Patient Health Questionnaire 9-Item).

	Four Mountains Task	Spatial Configuration Task	Mental Rotation Task	Path Integration Task
State-Trait Anxiety Inventory (STAI)	168	207	166	161
Patient Health Questionnaire (PHQ-9)	169	209	168	162

**Table 2:** Correlation between the State-Trait Anxiety Inventory (STAI) Score and the Four Mountains Task, the Spatial Configuration Task, the Mental Rotation Task, and the Path Integration Task.

		Four Mountains Task	Spatial Configuration Task	Mental Rotation Task	Path Integration Task
**State-Trait Anxiety Inventory (STAI) Score**	Pearson Correlation	−0.044	−0.042	−0.035	0.010
	Sig. (2-tailed)	0.570	0.549	0.653	0.903
	N	168	207	166	161

**Table 3:** Correlation between the Patient Health Questionnaire (PHQ-9) Score and the Four Mountains Task, the Spatial Configuration Task, the Mental Rotation Task, and the Path Integration Task.

		Four Mountains Task	Spatial Configuration Task	Mental Rotation Task	Path Integration Task
**Patient Health Questionnaire (PHQ-9) Score**	Pearson Correlation	−0.069	0.013	−0.029	−0.191 *
	Sig. (2-tailed)	0.374	0.857	0.709	0.015
	N	169	209	168	162

* Correlation is significant at the 0.05 level (2-tailed).

## Data Availability

Data are contained within the article.
